# Acute Effects of High-Intensity Interval and Moderate-Intensity Continuous Exercise on GLP-1, Appetite and Energy Intake in Obese Men: A Crossover Trial

**DOI:** 10.3390/nu10070889

**Published:** 2018-07-12

**Authors:** Victor A. F. Matos, Daniel C. Souza, Victor O. A. Santos, Ítalo F. Medeiros, Rodrigo A. V. Browne, Paulo R. P. Nascimento, Cristiane S. R. Marinho, Alexandre C. Serquiz, Eduardo C. Costa, Ana Paula Trussardi Fayh

**Affiliations:** 1 Graduate Program in Physical Education, Federal University of Rio Grande do Norte, Natal 59078-970, Brazil; victormattos_@hotmail.com (V.A.F.M.); daniel_souza86@hotmail.com (D.C.S.); victoroliveira.ufrn@hotmail.com (V.O.A.S.); ecc@ufrnet.br (E.C.C.); 2 Department of Nutrition, Federal University of Rio Grande do Norte, Natal 59078-970, Brazil; italofreire@icloud.com; 3 Graduate Program in Health Sciences, Federal University of Rio Grande do Norte, Natal 59078-970, Brazil; rodrigobrowne@ufrn.edu.br (R.A.V.B.); alexandreserquiz@gmail.com (A.C.S.); 4 Institute of Tropical Medicine, Federal University of Rio Grande do Norte, Natal 59078-970, Brazil; prpn1987@gmail.com; 5 Health Science College of Trairi, Federal University of Rio Grande do Norte, Santa Cruz 9200-000, Brazil; cristiane_ramos@hotmail.com

**Keywords:** obesity, high intensity interval exercise, hunger, compensation, gastrointestinal hormones, energy intake, T0, T30 and T90 min, respectively

## Abstract

This study investigated the effect of high-intensity interval (HIIE) and moderate-intensity continuous exercise (MICE) on glucagon-like peptide 1 (GLP-1), appetite and energy intake (EI) in obese men. In a randomized crossover trial, 12 participants (28.4 ± 2.6 years, 35.5 ± 4.5 kg/m^2^, 39.8 ± 2.2% body fat) performed: (I) Control (CON, no exercise); (II) MICE (20 min, 70% of maximal heart rate) and (III) HIIE (10 × 1 min at 90% of maximal heart rate with 1 min recovery). GLP-1 and appetite were assessed at: (I) PRE: pre-exercise; (II) POST: immediately post-exercise; (III) POST-1 h: 1 h post-exercise. EI was assessed after an *ad libitum* meal offered 1 h post-exercise and over 24 h. There was a significant time × condition interaction for GLP-1 (*p* = 0.035). Higher GLP-1 levels in MICE vs. CON (*p* = 0.024) and a trend for HIIE vs. CON (*p* = 0.069) POST-1h was found. Hunger was reduced immediately post-HIIE compared to CON (*p* < 0.01), but was not sustained POST-1 h (*p* > 0.05). EI did not differ between the sessions 1 h post-exercise or over 24H (*p* > 0.05). In summary, although MICE increased GLP-1 levels POST-1h and HIIE induced a transient reduction in hunger, both exercise protocols did not impact EI in obese men.

## 1. Introduction

Obesity is characterized as an excessive fat accumulation and is considered one of the main factors responsible for an increased incidence of metabolic dysfunctions, such as insulin resistance and chronic low-grade inflammation [1]. The prevalence of obesity has increased considerably in recent years, resulting in a global public health problem [2]. Therefore, the implementation of weight-loss-promoting practices such as physical exercise and diet are important cornerstones to reduce cardiometabolic risk associated with obesity [3,4].

Appetite responses should be considered for weight loss, since appetite and food intake are regulated at the physiological level in the neuroendocrine system, in which gastrointestinal hormones (including GLP-1) interact with the central nervous system, mediating the episodes of hunger and satiety [5]. More specifically, GLP-1 is an anorexigenic hormone secreted by Intestinal L cells which influence gastric emptying, insulin secretion and satiety signaling [6].

Therefore, evidence suggests that a single session of aerobic exercise may promote changes in gastrointestinal hormones, inducing a transient suppression on appetite and energy intake; an effect known as exercise-induced anorexia (EIA) [7,8], and the magnitude of these responses may occur in an intensity-dependent manner [9,10,11]. In addition, a recent meta-analysis showed a mean effect for acute moderate to vigorous exercise to increase GLP-1 levels in normal-weight individuals [12], but these findings cannot be applied to an obese population.

Recently, high intensity interval exercise (HIIE) has been considered an alternative approach to traditional moderate-intensity continuous exercise (MICE) training to improve cardiometabolic health in overweight and obese individuals [13,14]. HIIE is characterized by relatively short bouts of high-intensity exercise (i.e., 80–100% of maximal heart rate) interspersed by recovery periods [14]. This modality can reduce waist circumference, resting heart rate and systolic and diastolic blood pressure and increase cardiorespiratory fitness in overweight and obese individuals [13,14]. However, the effects of HIIE on appetite responses and energy intake in obese individuals are less known and controversial. For example, SIM et al. [9] showed that HIIE (30 min, 60 s at 100% VO_2peak_ and 240 s at 50% VO_2peak_) and sprint interval exercise (30 min, 15 s at 170% VO_2peak_ and 60 s at 32% VO_2peak_) suppressed energy intake after exercise in overweight-obese men. On the other hand, Martins et al. [15] found no difference in appetite perception and energy intake after MICE (30 min at 70% HR_max_) and HIIE (20 min, 8 s “all out” and 12 s at active recovery) in obese subjects, which makes these findings still lacking.

Therefore, the purpose of this study was to determine the effect of different exercise intensities (MICE and HIIE) on GLP-1, appetite and energy intake in obese sedentary males. Our initial hypothesis was that a greater EIA (increased satiety and GLP-1 concentration, reduced hunger and energy intake) may occur in HIIE compared to MICE.

## 2. Material and Methods

### 2.1. Study Design and Subjects

This is a randomized controlled crossover trial involving obese males (Body Mass Index-BMI: 30–39.9 kg/m^2^), aged between 22 and 41 years, previously sedentary and not using any drugs. Active smokers and patients with overt hypothyroidism, diabetes mellitus, grade 3 obesity (BMI ≥40 kg/m^2^), arterial hypertension, anemia, active infection or cancer were excluded. Participants were recruited from an invitation disclosed in university settings, e-mails and online social networks in the city of Natal, Brazil.

For sample size calculation, a statistical power analysis a priori was conducted considering the analysis of variance being used in the GLP-1 concentration (two-way repeated measures ANOVA), with an estimated effect size of 25% (considered middle-sized), statistical power 1-β of 80%, and an alpha of 5%. The sample size required for the study was 12 participants (G*Power, version 3.1.9.2; Institute for Experimental Psychology in Dusseldorf, Germany).

The present study was conducted in accordance with the Declaration of Helsinki, and all procedures involving human subjects/patients were approved by the Research Ethics Committee of Federal University of Rio Grande do Norte (Protocol No. 976.389/2015). Written informed consent was obtained from all subjects. The trial has been registered in a Brazilian clinical trials platform (ReBEC, No. RBR-62kr6f).

### 2.2. Procedures

**Figure 1:**
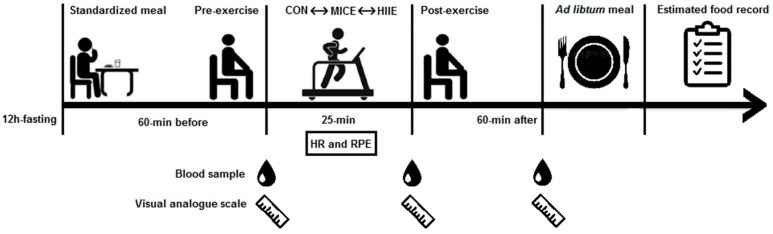
Flowchart of experimental sessions. Standardized meal after 12 h fasting; 60 min resting prior to exercise; exercise session; 60 min of recovery; *Ad libitum* meal; Estimated food record. HR: heart rate; RPE: Rate of perceived exertion.

The volunteers attended the laboratory at 8 a.m. after 12 h of overnight fasting and remained there for the next 3 h. All participants performed a total of four visits until completing the survey. At the first visit for familiarization and baseline measures, fasting blood sampling, resting blood pressure [16] by oscillometric method (HEM-7200, Omron, Hoffman States, IL, USA) and anthropometric assessment were performed, followed by an incremental test (RT250, Movement^®^, Pompeia, Brazil) to determine the maximal treadmill velocity (MTV) and maximum heart rate (HR_max_) in the experimental sessions. On the following visits, the volunteers randomly underwent three experimental conditions: (1) MICE (20 min, 70% HR_max_); (2) HIIE (10 × 60 s, 90% HR_max_ + 60 s, 30% HR_max_); or (3) control session without exercise (CON). Each session was interspaced by one week. Participants registered their food consumption 24 h before the first experimental session and were instructed to consume the same foods and not exercise 24 h prior to each subsequent session. Figure 1 illustrates the flowchart of experimental sessions.

### 2.3. Anthropometric and Body Composition Assessment

The following anthropometric measurements were assessed: body mass and height for calculating of BMI and classification of nutritional status according to WHO [17]. Body mass was measured on a digital scale (BC 553, Tanita^®^, Arlington Heights, IL, USA) with a maximum capacity of 150 kg and precision of 100 g, with the individual barefoot and wearing light clothes. A portable stadiometer with an accuracy of 1 mm (Personal Caprice Portatil, Sanny^®^, São Bernardo do Campo, SP, Brazil) was used to measure height with the individuals in the Frankfurt position. Double-energy X-ray absorptiometry (DEXA) was also analyzed (GE, Medical Systems, Chicago, IL, USA), and the participants were instructed to avoid diuretics and caffeinated beverages the day before the evaluation.

### 2.4. Biochemical Measurements

In the first visit, the following biochemical measurements were performed to characterize the participants after 12 h fasting: glucose, total cholesterol, high-density lipoprotein (HDL-cholesterol), low-density lipoprotein (LDL-cholesterol), very low-density lipoprotein (VLDL-cholesterol) and triglycerides. Biochemical markers were determined by colorimetric methods, using commercially specific kits (Doles^®^ kit, São Paulo-SP, Brazil). LDL-cholesterol was calculated by the Friedewald formula [18] and VLDL-cholesterol was calculated considering triglycerides dividing by 5. During the experimental sessions, total GLP-1 was assessed at three time points: PRE: pre-exercise, 1 h after standardized meal; POST: immediately post-exercise; POST-1 h: 1 h post-exercise. GLP-1 was assessed by enzyme immunoassay with specific kits (Sigma Aldrich^®^, St. Louis, MO, USA). During each blood collection, 10 mL were withdrawn from a vein in the antecubital region by a trained professional. The blood was subsequently centrifuged for 15 min at 3600 revolutions per minute. The serum was separated into 200 microliter aliquots and stored at −80 °C for further analysis. For lactate determination after exercise, the tip of the individual’s finger was sanitized with a 70% alcohol solution and then pierced with a disposable lancet immediately after HIIE and MICE. Approximately 25 µL of blood were collected and analyzed on a specific portable monitor (Accutrend Plus^®^, Roche, Switzerland).

### 2.5. Maximal Graded Exercise Test

The participants performed a warm-up on a treadmill (RT250, Movement^®^, Pompeia, Brazil) at a speed of 2.0 km/h for three minutes. Then they started the incremental test at a speed of 3.0 km/h and increments of 1.0 km/h every minute until voluntary exhaustion. The MTV was considered as the highest velocity sustained by a full stage of one minute [19,20,21]. Heart rate (HR) was monitored during the test using a HR monitor (RS800CX, Polar^®^, Kempele, Finland) and recorded at the end of each minute. The highest HR value observed during the test was considered as the HR_max_. Subjective perceived exertion (SPE) was also monitored during the test and recorded at the end of each minute according to the Borg scale 6–20 [22]. The test end was determined by the presence of at least one of the following criteria: (i) HR ≥ 100% estimated for age; (ii) SPE > 18; or (iii) when participants voluntarily stopped [23].

### 2.6. Standardized Meal

After a 12-h fasting period and prior to each experimental session, the participants consumed a standardized liquid meal (Mass Titanium^®^, Max Titanium, Matão, Brazil) 60 min prior to the exercise sessions and control. The commercial product was powdery, and it was reconstituted in water to provide 4.5 kcal × body weight (kg) to each participant. According to the manufacturer, each 100 g of powder has 377 kcal (87.5% of carbohydrates, 11.2% of proteins and 1.3% of lipids). The offered meal met the nutritional recommendations for pre-exercise calorie amount and macronutrient distribution [24].

### 2.7. Experimental Sessions

The MICE session consisted of 20 min at 65–75% peak HR performed continuously, which is the exercise intensity recommended as moderate by the American College of Sports Medicine physical activity guidelines [25]. The participants performed a 3 min warm-up at 4 km/h before both exercise sessions, and a 2 min cool-down at the same speed after the exercise sessions. Low-volume HIIE was performed in a 1:1 “effort-recovery”. The participants performed 10 × 1 min work bouts at 90% of their individual MTV reached on the maximal graded exercise test, interspersed by 1 min of active recovery at 30% of MTV (i.e., slow walking). This low-volume HIIE model on a treadmill was previously published by our research group [18,19,20]. HR was continuously recorded throughout the exercise sessions (Polar Electro^®^, Oy, Finland). In addition, whole-body rating of perceived exertion (RPE) was assessed using the RPE 6–20 Borg scale [22] during the last 10 s of each minute during both HIIE and MICE. In the control session, the participants remained in a seated position for 25 min and they were allowed to read and use electronic devices such as a tablet, computer or smartphone. The participants were instructed to abstain from moderate and vigorous physical activity and alcohol intake for the 24 h before the experimental sessions, and to maintain their normal eating habits and a good sleeping pattern.

### 2.8. Subjective Appetite Perception

Subjective appetite perception was performed in experimental conditions and control at three time points: PRE, POST and POST-1 h. The appetite perception measurements were recorded through a visual analogue scale that involves four aspects: hunger, satiety, fullness and perspective of food consumption (PFC). This scale is valid and reproducible for assessing appetite perception [26,27]. The scale consists of four 100-mm horizontal lines which point out the appetite perception, with little or no perception on the right side and the opposite perception on the left extremity. Participants were instructed to mark a vertical line at the point where their perception approached.

### 2.9. Ad Libitum Meal

The *ad libitum* meal was offered one hour after the experimental sessions in a reserved room. Food was offered as a “buffet” and participants were invited to eat “until they felt comfortably satisfied”. The following food options were part of this buffet: apples, bananas, toast, natural yogurt, potato chips, chocolate, fruit juice, boiled eggs, jam and butter (Appendix A). The food options were weighed before and after consumption and dietary intake was performed with food analysis software (Dietwin Profissional^®^ version 2016, Porto Alegre, RS, Brazil). The selected foods presented in the *ad libitum* meal were chosen according to the Brazilian population’s food guide [28], which establishes guidelines for a healthy and adequate diet considering social, economic and cultural aspects of each region of Brazil.

### 2.10. Estimated Food Record

An estimated food record was applied to evaluate energy intake over 24 h. Therefore, the participants described all foods consumed and their respective amounts on forms throughout the day in each experimental session according to standard nutritional assessment protocols [29]. Detailed data about food preparation methods, ingredients used in mixed dishes, and the brand name of commercial products may be required according to the research question. The amounts of each food consumed are estimated in reference to a common size container (e.g., bowls, cups, and glasses), and the participants also had a digital precision scale (Thinox, Plenna^®^, São Paulo, Brazil) to weigh and describe the amount of food consumed. The dietary intake analysis was performed with food analysis software (Dietwin Profissional^®^ version 2016).

## 3. Statistical Analysis

Data normality was verified by the Shapiro-Wilk test, asymmetry and kurtosis. Parametric descriptive data were presented in mean and standard deviation (SD) or standard error (SE), and the non-parametric data in median and 25th and 75th percentiles. Dependent variables, HR and lactate were compared during experimental sessions through the Student’s *t*-test. Two-way repeated measures analysis of variance (ANOVA) followed by Bonferroni’s post hoc was used to verify the condition x time interaction for dependent variables (hunger, satiety, fullness, PFC, total GLP-1 and delta GLP-1 variation). Repeated measures ANOVA or Friedman’s test was applied in the comparison between experimental conditions and control for dependent variables (energy intake, carbohydrates, proteins, lipids). Sphericity hypothesis was verified by the Mauchly test, and the degrees of freedom were corrected by Greenhouse–Geisser estimates when violated. The effect size of the variances was calculated by the partial square eta (*η*^2^*p*). The significance level was adopted at *p* < 0.05. All statistical procedures were performed using SPSS for Win v.20.0 (Statistical Package for Social Sciences, Chicago, IL, USA).

## 4. Results

**Figure 2:**
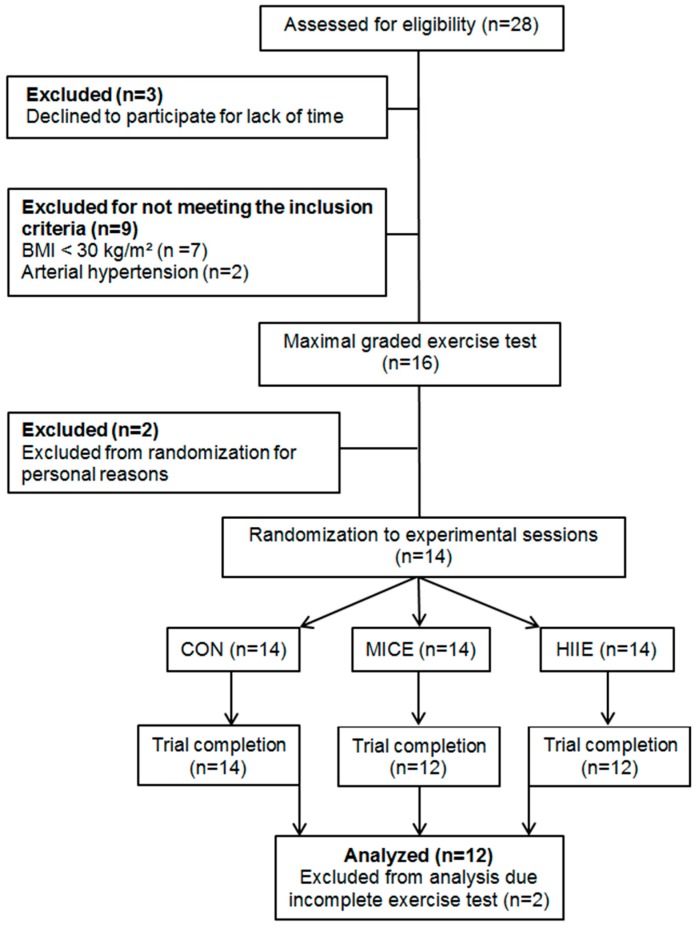
Selection flowchart of participants.

Twenty-eight (28) subjects initially answered the invitations and were assessed for eligibility criteria, but only 16 subjects started the study protocol. After assessment for eligibility and randomization for experimental sessions, twelve individuals completed the trial. Figure 2 summarizes the selection process of study volunteers. The sample characteristics are described in Table 1. Biochemical and hemodynamic markers were within normal range, suggesting an absence of cardiometabolic alterations.

Upon characterizing experimental sessions, higher HR values for HIIE compared to MICE (83.7 ± 6.8% HR_max_ vs. 70.5 ± 1.1% HR_max_; *p* = 0.001) were observed, and a higher lactate concentration in HIIE compared to MICE (12.5 ± 2.6 mmol/L vs. 4.9 ± 1.4 mmlol/L; *p* = 0.001) after exercise sessions was also observed, which indicates a more pronounced cardiovascular demand and metabolic stress for HIIE.

**Figure 3:**
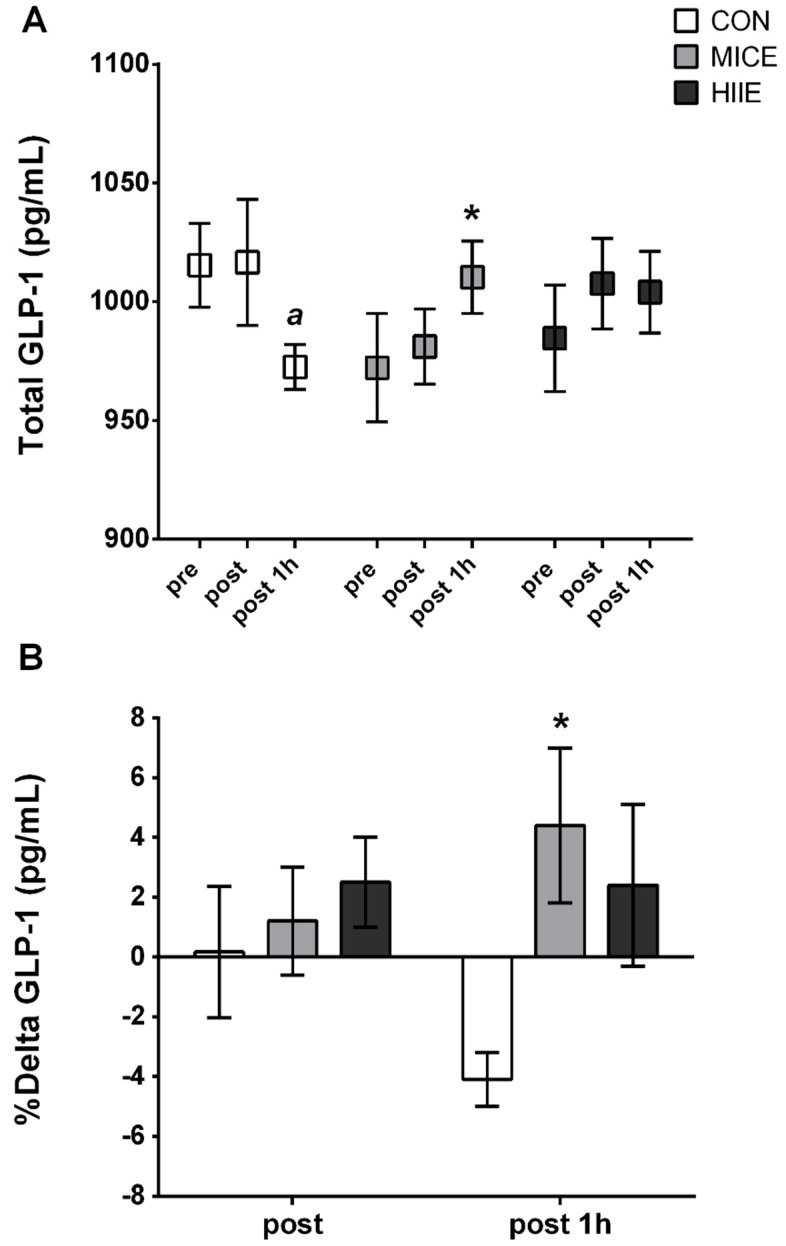
(**A**) Total GLP-1 in experimental and control sessions; (**B**) Delta GLP-1 post and post 1 h. Results expressed as mean ± SE. *^a^* Significantly different from pre (*p* < 0.05); * Significantly different from CON at same time point (*p* < 0.05).

GLP-1 showed a significant effect of time x condition interaction (*F*(4, 44) = 2.874, *p* = 0.035, *η*^2^*p* = 0.223, power = 0.73), while the Bonferroni’s post hoc test indicated higher GLP-1 concentration in MICE vs. CON (*p* = 0.024) and a trend for higher levels in HIIE vs. CON (*p* = 0.069) POST-1 h. Additionally, a significant intra-condition decrease in GLP-1 was only observed in CON (PRE vs. POST-1 h, *p* = 0.006) (Figure 3A). Similarly, there was a significant effect of time × condition interaction in delta GLP-1 (*F*(2, 20) = 3.607, *p* = 0.046, *η*^2^*p* = 0.265, power = 0.60). Therefore, this variable was increased in MICE vs. CON (*p* = 0.038) POST-1 h. Moreover, delta GLP-1 pointed to a decrease during the session in the CON (post vs. post 1 h; *p* = 0.060) and presented an increase in the MICE (post vs. post 1 h, *p* = 0.063) (Figure 3B).

Regarding subjective appetite perception, there was a significant time x condition interaction for hunger (*F*(4, 44) = 5.19, *p* = 0.002, *η*^2^*p* = 0.321, power = 0.95). Thus, Bonferroni’s post hoc test revealed that POST exercise had reduced hunger in HIIE vs. CON (*p* = 0.009). However, an intra-condition of increased hunger was observed over the session for HIIE (PRE vs. POST-1 h, *p* = 0.042; POST vs. POST 1H, *p* < 0.001) and in CON (PRE vs. POST 1H, *p* = 0.023).

**Figure 4:**
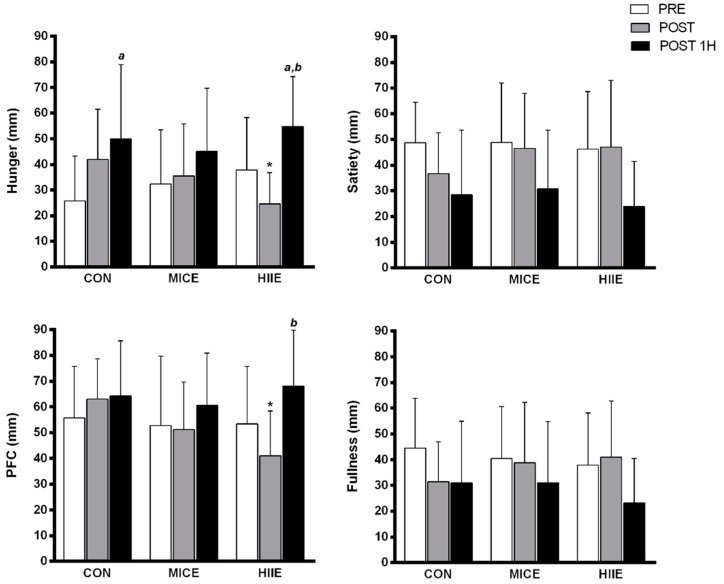
Subjective appetite perception at different exercise conditions and control. Values presented in mean ± SD. *^a^* Significantly different from PRE (*p* < 0.05); *^b^* Significantly different from POST; * Significantly different from CON at same time point (*p* < 0.05).

Furthermore, a significant time x condition interaction also was found in PFC (*F*(4, 44) = 3.993, *p* = 0.008, *η*^2^*p* = 0.266, power = 0.88). Accordingly, POST exercise reduced PFC was shown in HIIE vs. CON (*p* = 0.005). Nonetheless, a significant intra-condition of increased PFC was observed in HIIE during the session (POST vs. POST-1 h, *p* = 0.003). No significant time x condition interactions were observed for satiety (*F*(4, 44) = 1.58, *p* = 0.159, *η*^2^*p* = 0.126, power = 0.45) or fullness (*F*(4, 44) = 2.09, *p* = 0.098, *η*^2^*p* = 0.160, power = 0.58) (Figure 4).

There was no significant difference in EI between conditions in *ad libitum* meal (*F*(2, 22) = 0.73, *p* = 0.489, *η*^2^*p* = 0.063, power = 0.16) and 24 h (*F*(2, 22) = 0.51, *p* = 0.606, *η*^2^*p* = 0.045, power = 0.13). Similarly, macronutrients were not modified in the *ad libitum* meal for carbohydrates (*F*(2, 22) = 0.582, *p* = 0.567, *η*^2^*p* = 0.050, power = 0.14); proteins (*F*(2, 22) = 0.856, *p* = 0.439, *η*^2^*p* = 0.072, power = 0.18); or lipids (*F*(2, 22) = 0.504, *p* = 0.611, *η*^2^*p* = 0.044, power = 0.12). No differences were also observed in macronutrient intake over 24 h in carbohydrates (*χ*^2^ (2) = 1.16, *p* = 0.558); protein (*χ*^2^ (2) = 0.167, *p* = 0.920); or lipids (*χ*^2^ (2) = 3.16, *p* = 0.205) (Table 2).

## 5. Discussion

The main findings of the present study did not confirm our hypothesis that HIIE may induce EIA. In summary, MICE and HIIE presented higher or sustained GLP-1 concentration through experimental sessions compared to CON, and a decreased GLP-1 level was only observed in CON. Additionally, there was no difference in subjective appetite perception at different exercise intensities in obese men, except for a reduction in hunger and PFC after exercise in HIIE vs. CON, which was not sustained after 1H. Finally, the different exercise intensities did not suppress energy intake in obese men, although there was no subsequent increase up to 24 h after experimental sessions.

Initially, the present study found higher GLP-1 levels up to 1h after exercise sessions in comparison to the CON session, as well as a reduced GLP-1 concentration compared to the experimental session only in CON. Possibly, the significant reduction in GLP-1 in the CON session is due to the absence of exercise in this group, once that previous results have described the effect of exercise on increased GLP-1 responses [12,30,31]. At this point, it is suggested that increased GLP-1 levels occur through an elevation in catecholamines and circulating free fatty acids, which stimulates the intestinal l-cells during exercise [11], and which may explain our results. However, the studies that found improvements in GLP-1 in obese subjects used high-volume moderate to vigorous exercise protocols (60 min, 50–70% VO_2max_) [30,31] or maximal protocols of low-volume HIIE involving “all out” effort [15,32].

It is important to highlight how a brief session of MICE (20 min) was able to increase GLP-1 levels in sedentary obese males, but in contrast to previous studies [15,32], we did not observe a significant difference between exercise conditions. At this point, we could expect higher GLP-1 levels after HIIE, given that higher intensity exercises provoke a greater sympathetic activation, which may result in larger release of this peptide [11,12,13,14,15,16,17,18,19,20,21,22,23,24,25,26,27,28,29,30,31,32,33]. Supporting these findings, these previous studies were performed with HIIE maximal protocols with “all out” bouts, resulting on higher GLP-1 levels [15,32]. Therefore, it’s possible that the low volume-submaximal characteristic of HIIE used in our study was not enough to produce such responses.

Interestingly, a more pronounced effect was found for MICE when a higher delta GLP-1 was revealed 1 h after exercise session compared to CON (+4.4% vs. −4.1%, respectively), which indicates that GLP-1 levels continued increasing after exercise. Nevertheless, the different response in GLP-1 during exercise sessions compared to CON was not accompanied by clinical changes (i.e., increased satiety, reduced energy intake). Similar inconsistences also were found in previous studies with overweight and obese subjects [15,31]. This acute change was possibly not enough to impact the clinical response since the desire and the decision to eat are not exclusively due to hormonal changes [34].

In agreement with our findings about subjective appetite perception, Alkahtani et al. [35] found no difference in hunger and satiety between HIIE in a cycle ergometer (15 s at 85% VO_2max_ + 15 s active recovery) vs. MICE (30 min, Fat_max_) in overweight/obese individuals. However, in the present study, appetite perception was also performed 60 min after the experimental sessions, which enabled observing no subsequent increases in appetite perception up to 1H after exercise sessions. In addition, a previous study conducted by our research group observed no differences for subjective appetite perception in MICE (20 min at 65% HR_max_) vs. HIIE (10 × 60 s at 90% HR_max_) in overweight subjects up to 40 min after exercise session [36]. Thus, it’s possible that obese males presented an appetite perception response similar to overweight individuals when submitted to MICE and HIIE protocols, without present subsequent compensatory increases in appetite perception [9,35,36].

On the other hand, we found significantly reduced hunger and PFC in HIIE vs. CON immediately after exercise, but this response was not sustained after 1H, which suggests a transient effect. These findings are in agreement with King et al. [7] who observed appetite suppression after 30 min of exercise (70% VO_2max_); and Broon et al. [8] who found a significant reduction in hunger after treadmill running (72% of VO_2max_). In this same study, a positive correlation between reduced hunger and suppressed ghrelin levels (*r* = 0.69, *p* < 0.01) was observed. Therefore, a possible reason explaining the acute reduced hunger and PFC is increased blood flow directed to muscle tissue during exercise which consequently decreased to the gastrointestinal area, resulting in a lower activation of P1/D1 ghrelin producing cells in the stomach [9,11].

Since the appetite perception variables were little affected in that manner that transient reduction in hunger and PFC were observed only in HIIE, a concomitant or small reduction in subsequent energy intake could be estimated; however, this present study found no significant differences for the *ad libitum* meal (1 h post exercise) or throughout the day (24 h) between experimental conditions and control. Different from our findings, Sim et al. [9] revealed a significant reduction in energy intake after HIIE (60 s to 100% VO_2peak_ + 240 s to 50% VO_2peak_) and supramaximal exercises (15 s to 170% of VO_2peak_ + 60 s to 32% of VO_2peak_) for the *ad libitum* meal and 24 h in 17 overweight individuals. Nonetheless, the performed exercise intensity was much higher compared to the intensities adopted in the present study, and at levels that would be difficult to sustain in obese subjects (which may explain the different results); also, the sample size adopted in our study may not be appropriate to identify any possible significant differences in these variables.

On the other hand, it is important to clarify that both exercise sessions in the present study did not exceed the energy intake compared to control for the *ad* *libitum* meal or throughout the day. In agreement with the present findings, Martins et al. [15] also did not find significant differences in subsequent energy intake throughout the day in MICE (70% HR_max_) or HIIE (8 s all out +12 s at 20–30 rpm) with obese subjects. These findings are similar to a previous meta-analysis in normal-weight subjects, where no increased energy intake was observed after low to vigorous exercise intensity (36–81% VO_2max_) [37]. Therefore, it is suggested that obese males present an absence of compensatory increased energy intake after both exercise conditions.

Regarding the strengths of present study, we offered a standardized meal before exercise sessions and CON. This measure is important to prevent individual changes in appetite between conditions. Additionally, the energy intake measurements were performed one hour after exercise (*ad libitum* meal) and throughout the day (24 h) by an estimated food record, which permitted food consumption in an external environment. However, the study has some limitations. The exercise protocols (MICE and HIIE) were matched by time (20 min/session) and not by caloric expenditure, which did not allow for observing differences in expended energy. In addition, an inevitable limitation refers to the method for estimating 24 h food consumption, which depends on the respondent’s integrity. In order to minimize this limitation, a well-trained interviewer was selected to minimize recall bias. Finally, despite the instructions for the participants to maintain their eating habits and sleeping habits 24 h before the experimental sessions and to avoid moderate and vigorous physical activity and alcohol intake, compliance with these instructions was not assessed.

From a practical point of view, the sustained exercise-induced elevation in GLP-1 may point to enhanced satiety signaling for a prolonged period in obese males. Moreover, the current physical activity guidelines recommend that adults should perform 30–60 min of moderate-vigorous exercise on most days of week [24], however, almost all individuals with obesity do not meet this recommendation [38]. Therefore, our study showed that for inactive individuals with obesity both exercise protocols (HIIE and MICE) with a reduced time commitment were able to increase GLP-1 levels without an increase in subsequent energy intake. Thus, considering the individual preferences and motivations, both low-volume HIIE and MICE could be considerable for this population.

For future studies, we suggest increasing the follow-up time for post-exercise GLP-1 levels, as well as to measure additional appetite-related hormones such as acylated ghrelin, cholecystokinin and insulin. Furthermore, additionally investigating alternative exercise protocols (i.e., sprint interval exercise) would be of great value in helping to discuss the biochemical mechanisms of appetite control.

## 6. Conclusions

HIIE and MICE seem to elicit similar effects on GLP-1, increasing its levels up to 1 h post-exercise in obese men. However, despite a transient reduction in hunger immediately post-HIIE, both exercise protocols do not change energy intake 1-h or 24 h post-exercise.

## Figures and Tables

**Table 1:** Characteristics of the sample (*n* = 12).

Title	Mean ± SD
Age (years)	28.4 ± 2.6
Height (cm)	177 ± 7
Body weight (kg)	109.0 ± 17.1
Body mass index (kg/m^2^)	35.5 ± 4.5
Fat free mass (kg)	65.7 ± 10.7
Body fat (%)	39.8 ± 2.2
Fasting glucose (mg/dL)	98.6 ± 25.2
Total cholesterol (mg/dL)	198.1 ± 25.9
HDL-cholesterol (mg/dL)	42.7 ± 2.3
LDL-cholesterol (mg/dL)	131.5 ± 27.1
VLDL-cholesterol (mg/dL)	24 ± 7.4
Tryglicerides (mg/dL)	119.9 ± 37.1
**Physical Activity Level**	
Walking (min/week)	16.9 ± 21.3
Moderate activity (min/week)	10.8 ± 15
Vigorous activity (min/week)	0
Sitting time (h/day)	9.7 ± 3.3

**Table 2:** Energy intake and macronutrients in *ad libitum* meal and throughout the day for the experimental sessions and control.

Title	CON	MICE	HIIE
Energy Intake (kcal)			
*Ad libitum*	766 ± 189	713 ± 196	761 ± 243
24 h	2813 ± 462	2737 ± 535	2665 ± 435
Carbohydrate (g)			
*Ad libitum*	91.3 ± 32.3	85.4 ± 27.9	91.7 ± 31.1
24 h	399.9 (371–465)	380.2 (327–427)	362.6 (311–455)
Protein (g)			
*Ad libitum*	26.9 ± 7.9	24.9 ± 6.9	25.3 ± 8.9
24 h	99.2 (88–143)	108.2 (90–134)	112.9 (102–117)
Lipids (g)			
*Ad libitum*	32.5 ± 8.0	30.3 ± 11.1	32.5 ± 11.5
24 h	86.9 (70–111)	78.5 (59–93)	81.1 (68–92)

Values presented in mean ± SD or median (interquartile range 25–75). CON: Control session; MICE: Moderate intensity continuous exercise; HIIE: high intensity interval exercise. No statistically significant difference was observed between conditions.

## Appendix A

**Table A1:** Food Items Provided in the *Ad Libitum* Buffet Meal.

Food	Amount (g or mL)	Energy (Kcal)	CHO (g)	PTN (g)	LIP (g)
Apple	120 g	82.8	19.9	0.2	0.2
Banana	180 g	156.4	36.5	2.2	0.2
Fruit Juice	200 mL	107.6	26	0.9	0
Whole Yogurt	170 g	126.5	9.1	6.8	7
Potato chips	27 g	153	13	1.7	10.5
Chocolate	40 g	191.2	24	2.2	9.6
Toast	20 g	77	13.2	3.1	1.3
Boiled egg	150 g	211.7	0.9	20	14.3
Fruit jelly	30 g	74.3	18.5	0	0
Butter	20 g	148.7	0	0.1	16.5
Total	957	1329	161.2	37.2	59.6
Total (% macronutrients)	-	-	48.5	11.2	40.3

CHO: carbohydrate, PTN: protein, LIP: lipid.
